# Describing the content of trial recruitment interventions using the TIDieR reporting checklist: a systematic methodology review

**DOI:** 10.1186/s12874-024-02195-5

**Published:** 2024-04-08

**Authors:** Natasha Hudek, Kelly Carroll, Seana Semchishen, Shelley Vanderhout, Justin Presseau, Jeremy Grimshaw, Dean A. Fergusson, Katie Gillies, Ian D. Graham, Monica Taljaard, Jamie C. Brehaut

**Affiliations:** 1 Clinical Epidemiology Program, Ottawa Hospital Research Institute, 501 Smyth Road, Box 201B, Ottawa, ON K1H 8L6 Canada; 2 School of Epidemiology and Public Health, University of Ottawa, Ottawa, ON Canada; 3 Health Services Research Unit, University of Aberdeen, Foresterhill, Aberdeen UK

**Keywords:** Reporting, Recruitment interventions, Systematic review, Methodology review

## Abstract

**Background:**

Recruiting participants to clinical trials is an ongoing challenge, and relatively little is known about what recruitment strategies lead to better recruitment. Recruitment interventions can be considered complex interventions, often involving multiple components, targeting a variety of groups, and tailoring to different groups. We used the Template for Intervention Description and Replication (TIDieR) reporting checklist (which comprises 12 items recommended for reporting complex interventions) to guide the assessment of how recruitment interventions are described. We aimed to (1) examine to what extent we could identify information about each TIDieR item within recruitment intervention studies, and (2) observe additional detail for each item to describe useful variation among these studies.

**Methods:**

We identified randomized, nested recruitment intervention studies providing recruitment or willingness to participate rates from two sources: a Cochrane review of trials evaluating strategies to improve recruitment to randomized trials, and the Online Resource for Research in Clinical triAls database. First, we assessed to what extent authors reported information about each TIDieR item. Second, we developed descriptive categorical variables for 7 TIDieR items and extracting relevant quotes for the other 5 items.

**Results:**

We assessed 122 recruitment intervention studies. We were able to extract information relevant to most TIDieR items (e.g., brief rationale, materials, procedure) with the exception of a few items that were only rarely reported (e.g., tailoring, modifications, planned/actual fidelity). The descriptive variables provided a useful overview of study characteristics, with most studies using various forms of informational interventions (55%) delivered at a single time point (90%), often by a member of the research team (59%) in a clinical care setting (41%).

**Conclusions:**

Our TIDieR-based variables provide a useful description of the core elements of complex trial recruitment interventions. Recruitment intervention studies report core elements of complex interventions variably; some process elements (e.g., mode of delivery, location) are almost always described, while others (e.g., duration, fidelity) are reported infrequently, with little indication of a reason for their absence. Future research should explore whether these TIDieR-based variables can form the basis of an approach to better reporting of elements of successful recruitment interventions.

**Supplementary Information:**

The online version contains supplementary material available at 10.1186/s12874-024-02195-5.

## Background

Clinical trial recruitment is frequently challenging. Trial participation rates are consistently low across public and private research sectors and clinical specialties [1–4]. In one American study, 40% of National Cancer Institute-funded trials were discontinued, nearly half because of participation issues [5]. Similarly, 37% of trials funded by the UK National Institute of Health Research failed to meet participation targets [6]. There are substantial costs associated with low participation rates and trials failing to meet targets, including wasted resources, delayed innovation, potentially biased results, and ethical issues associated with exposing participants to risk without scientific gain [7].

Research to improve recruitment has yielded few generalizable lessons that can be widely employed to improve the success of trials. Research has focused on individual elements of an overall recruitment strategy; here we refer to these elements as ‘recruitment practices’. A Cochrane review on the topic reviewed 68 publications, including over 74,000 participants in total, to evaluate the effectiveness of many different recruitment practices. The authors found only two practices with clear evidence supporting their effectiveness to improve recruitment rates: open rather than blind trial designs result in greater participation, as does the use of telephone reminders (as opposed to postal reminders) for non-responders of an initial invitation [8]. Other recruitment practices have been tested in multiple studies, (e.g. patient information developed using bespoke user-testing, shortened patient information leaflets, financial incentives), but clear conclusions about their effectiveness have been impeded by the variable quality of included studies, variable reporting, potential methodological biases, or limited sample sizes [8, 9].

Overall strategies to optimize recruitment can constitute complex interventions that include many different elemental recruitment practices, may be targeted to various groups (e.g., potential participants, study recruiters), and may include varying levels of tailoring [10]. For example, the recruitment strategy for one trial involved potential participants receiving church-based educational sessions around clinical trial participation, led by trained faith leaders, that included discussions on the importance of community participation, myths about clinical trials, information on different clinical conditions, and newsletters providing study updates and clinical trial opportunities [11]. Such complex interventions pose a challenge to determining which specific practices led to the trial’s recruitment success, and which might generalize to other settings. In part, this is because of the lack of a widely used, coherent framework to help guide reporting of the important aspects of recruitment strategies. Without such a framework to guide reporting of these interventions and to support knowledge synthesis, it will continue to be difficult or impossible to determine the specific recruitment practices that successfully generalize, and the accumulation of knowledge around how to improve trial recruitment will be slowed.

The Template for Intervention Description and Replication (TIDieR) checklist was designed to provide guidance on 12 core elements to report with complex health care interventions [12]. The checklist and accompanying guide are intended to ensure the reporting of intervention elements that are considered essential for reviewers and editors, and for researchers replicating and building on these interventions. In order to gain a better understanding of which elements of recruitment interventions are most effective, we first need to develop a consistent method to describe and categorize these interventions. We selected the TIDieR checklist as a guide for this work, because the notion of recruitment as a complex intervention may help describe why some recruitment interventions are more effective than others, and the checklist can inform discussion of which complex intervention elements are important to report. We aimed to use the TIDieR checklist as a guide to (1) examine to what extent we could identify/extract information about each of the 12 items within recruitment intervention studies, and (2) observe additional detail for each item to describe useful variation among recruitment intervention studies.

## Methods

We used the Preferred Reporting Items for Systematic Reviews and Meta-Analyses (PRISMA) statement to support complete reporting of this study (Additional file 1, Appendix A) [13].

### Study selection

We sought to identify studies examining the effects of recruitment interventions on clinical trial participation. These are often randomized or quasi-randomized studies embedded within a clinical trial to observe actual effects on trial recruitment, or can be trials using hypothetical clinical trial scenarios to elicit participants willingness to participate.

#### Study sources

In order to reduce duplication of effort, we used previous work done by the Cochrane and Online Resource for Research in Clinical triAls (ORRCA) groups. The Cochrane review conducted a systematic search using multiple sources (the Cochrane Methodology Review Group Specialized Register in the Cochrane Library, MEDLINE, Embase, Science Criterion Index & Social Science Citation Index) up to and including publications from 2015. In order to further benefit from this cumulative knowledge base we used identical inclusion criteria to the Cochrane review; therefore, we included the same 68 studies from this review (for details see Treweek et al., (2018) [8]). Second, we updated this sample by searching the ORRCA database, which collects and indexes publications relevant to the field of recruitment and retention research for clinical trials on an ongoing basis [14].

#### Inclusion criteria

In line with the Cochrane review, we included all published articles with the following PICOS [15] inclusion criteria: *participants (P)* included potential trial participants including both patients and representative community samples; *interventions (I)* of interest included any intervention aimed at improving recruitment to the host trial of the publication; the *comparator (C)* could be either study recruitment methods as usual or another intervention aimed at improving recruitment; *outcomes (O)* of interest included the proportion or number of potential participants recruited to the host trial whether the decision was real or hypothetical ‘willingness to participate,’ and *study designs (S)* included both randomized and quasi-randomized trials of recruitment interventions. The host trial design also needed to be a randomized clinical trial.

#### Exclusion criteria

We excluded any articles where the host study being recruited to was a survey, observational cohort, or biobank study as these types of studies are considered lower risk for participants and may not present the same recruitment challenges as those recruiting to active trials.

To update the original set of articles included from Treweek et al. [8], we searched the ORRCA database on November 3rd, 2020 and again on August 11th, 2022 using the following search parameters:

Recruitment database only (excluded retention database).

Year: 2015 to 2022.

Evidence type: Randomized evaluation (including quasi-randomized trials).

Research methods: Nested randomized controlled trial.

Research outcome: number recruited or recruitment rate or willingness to participate or other or unknown.

Search results were de-duplicated and then screened at the abstract and full text level to ensure they met inclusion criteria. This screening was done by NH and reviewed by JCB or KC.

### Data extraction

Two of three coders extracted data from each study (NH as primary and KC or SS as secondary). Coders met regularly to discuss discrepancies between items in order to reach consensus, with a fourth coder (JCB) resolving any disagreements. We extracted data into a Microsoft Excel 2010 [16] capture form developed by the authors. This form was pilot tested on an initial set of 6 articles and revised for completeness and functionality.

For each publication, we extracted data for up to three study arms (control/comparator, intervention 1, intervention 2). The control/comparator arm was considered to be the least intense recruitment effort, or standard recruitment effort where not otherwise specified by the study authors. For studies with more than one intervention arm, we defined intervention 1 as a less intensive and intervention 2 as the more intensive intervention. We determined intensity of the intervention using several factors, including financial and time costs to the researchers and burden of time and effort on participants (e.g. phone call (intervention 2) vs. email reminders (intervention 1) vs. no reminder (comparator/control)). For studies with more than three arms, we only extracted data for the two arms considered the most intensive and least intensive based on the above criteria. For publications reporting more than one study, studies were coded separately in the extraction form provided they were independent studies (i.e. used distinct samples, randomization procedures, and interventions). For studies where the same intervention was applied to different samples, we selected the study where the sample most closely resembled the target population of the host trial.

The data extraction form included 8 sections. The current manuscript reports on three of these (background information, intervention details, and risks of bias); we will report data on the other five sections separately (use of shared decision-making, participant-centered involvement, theory use, use of behavior change techniques, and recruitment outcomes).

#### Background information

Background information included the study’s first author, year of publication, title, source, and country in which the study took place. We also extracted a brief description of the host trial (i.e. the trial into which the participants are being recruited), whether the decisions participants made would result in actual trial participation (real decision) or not (hypothetical decision), the trial phase of the host trial, clinical specialty of the host trial, recruitment trial participant age (mean or median age for full sample), and proportion of reported male/female participants.

We recorded host trial phase (i.e. Phase I, II, III, IV) based on author report, trial registry if provided, or failing either of these, inference from the descriptions provided. Behavioural interventions (e.g. smoking cessation, falls prevention) were included with phase III studies because they were not being evaluated for safety (phase I), efficacy (phase II), or at the surveillance stage (phase IV) but rather evaluating intervention effectiveness, analogous to phase III. This classification strategy is comparable to that of other work focusing on behavioural interventions [17, 18]. Studies that were recruiting into more than one trial were coded as ‘multiple trials/phases’. Studies where a phase could not be determined based on what was reported in the article were coded as ‘other.’

#### Intervention details (TIDieR)

We initially sought to evaluate the contents of recruitment intervention reporting according to TIDieR checklist items [12] by extracting information relevant to each item. For extracted information we then created descriptive variables to enable core aspects of the reporting of recruitment interventions to be described. Below, we outline the development process for these variables.

**TIDieR framework.** The TIDieR framework outlines 12 checklist items recommended for reporting the nature of complex interventions: (1) a name or phrase that describes the intervention, (2) rationale/theory/goal of the elements essential to the intervention, (3) physical or informational materials used in the intervention, (4) procedures/processes used in the intervention, (5) intervention provider, (6) modes of delivery of the intervention (e.g. face-to-face, phone, internet), (7) location where the intervention occurred, (8) the number of times and length of time the intervention was delivered, (9) tailoring or personalization made for intervention recipients, (10) modifications made to the intervention throughout the study, (11) whether adherence/fidelity was planned, and (12) actual adherence/fidelity reported [12].

**TIDieR reporting.** We assessed with what frequency we were able to extract information relevant to each of the 12 TIDieR items for each study. For each TIDieR item, we recorded whether information was extractable and with enough detail provided to understand methods relevant to the item.

**TIDieR descriptive variables.** Our second aim was to describe additional detail for each item to better understand useful variation among recruitment intervention studies. We characterised as many TIDieR items as possible as categorical variables that could be independently assessed by coders. Two coders (JCB, NH) went through 6 initial studies to develop an initial set of categories for each of the 12 TIDieR items. Subsequent consensus meetings (JCB, NH, and KC) centered on how TIDieR items should be defined in the context of recruitment trials, refining the codebook, and determining what should be extracted for the non-categorisable items.

The final set of descriptive variables included 7 items that could be categorized, and 5 that were collected as quotes. The categorical items were:

- rationale – whether study authors provide a clear link between what mechanism they believe will improve recruitment and the selected intervention;
- materials – two items: (1) categories for the ‘active’ ingredients of the intervention (e.g., video, modified documents, additional documents, incentives) and (2) an item indicating whether access to full materials were available;
- procedure - categorized using ORRCA categories from Treweek et al. [8] (pre-trial planning, changes during trial, modifications to consent process, modification to information given to potential participants, intervention targeted at recruiter/site, incentives, other);
- intervention provider – categorized as part of clinical care team, research team, or other;
- modes of delivery - categorized using the ontology developed by Marques et al. (2020) [19] (e.g., informational – human interactional, informational – printed material, environmental change);
- intervention location – categorized by where the intervention was ‘received’ (e.g., clinical setting, non-clinical setting); and
- frequency and duration – two items: (1) frequency categorized as once, twice, 3 or more times and (2) duration defined as the amount of time participants/intervention target spent receiving the intervention in minutes.

We could not categorize the remaining five items in ways that were reliably codable and reasonably concise, and so we opted simply to extract relevant quotes for these items: intervention description, tailoring, modifications, planned fidelity/adherence, and actual fidelity/adherence.

Several items were modified from the TIDieR item definitions provided by Hoffman et al. [12] in order to fit the included studies better. For example, item 2 recommends the description of any rationale, theory, or goal of the intervention. We extracted details on theory use in more detail separately, results that will be reported elsewhere. For the current paper, we focused on the intervention rationale. Since all studies provided some form of rationale, this item was defined as whether authors provided sufficient information to discern a clear link for ‘why’ they believed the intervention would improve recruitment; for some studies, raters determined that improving recruitment was not the primary goal of the study (e.g. goal was to improve participant understanding but also assessed recruitment outcomes) and therefore rated as ‘not applicable.’ As well, procedure (item 4), was renamed ‘intervention type’ and defined using the categories provided by ORRCA as listed in Treweek et al. [8]. In addition to capturing the different types of intervention materials (item 3) used, we also rated whether full materials were included in the publication (or as online links) because guidance is increasingly recommending the inclusion of full materials/data for publication. This variable was rated as ‘not applicable’ for studies with no materials relevant to the intervention.

For multi-arm trials, where two interventions were included in the extraction, the two intervention arms were combined when coding the TIDieR descriptive items. While this may lead to a perception that these studies had more complex interventions, most multi arm studies were simply a greater ‘dose’ of the same intervention in each arm and would not affect the TIDieR-related categories selected.

#### Risk of bias

The 2018 Cochrane review by Treweek et al. [8] assessed risk of bias (RoB) of the recruitment trials using the original 5-item version of the Cochrane risk of bias tool. In our review, we assessed bias using the most recent 22-item Cochrane RoB 2 tool for parallel trials [20]. This tool assesses risk of bias in 5 domains: (1) risk of bias from randomization, (2) risk of bias due to deviations from the intended intervention, (3) risk of bias due to missing outcome data, (4) risk of bias in measurement of the outcome, and (5) risk of bias in selection of the reported results. Assessment within each domain results in a domain judgement of low bias, some concerns, or high bias. Domain judgements are then aggregated into an overall bias rating where low risk indicates low bias in all five domains, some concerns indicate at least one domain with some concerns, and high bias indicates at least one domain with high bias, or multiple domains with some concerns. The tool also requires users to select an effect of interest for their studies (effect of assignment to intervention vs. effect of adherence to intervention). We defined the effect of interest as the effect of assignment to the intervention since trialists implementing recruitment interventions may have little control over intervention adherence (e.g. whether someone opens an email, letter, watches a video). The study team developed an Excel spreadsheet to include responses and justifications to each signaling question and automated the algorithms for domain and overall bias judgements. Risk of bias was assessed by two of three possible coders (NH, KC, SV) with all discrepancies resolved through consensus.

### Data analysis

We imported data into SPSS (version 28) for analysis. We calculated means, standard deviations, or frequencies for the demographic and TIDieR-related variables.

## Results

**Fig. 1:**
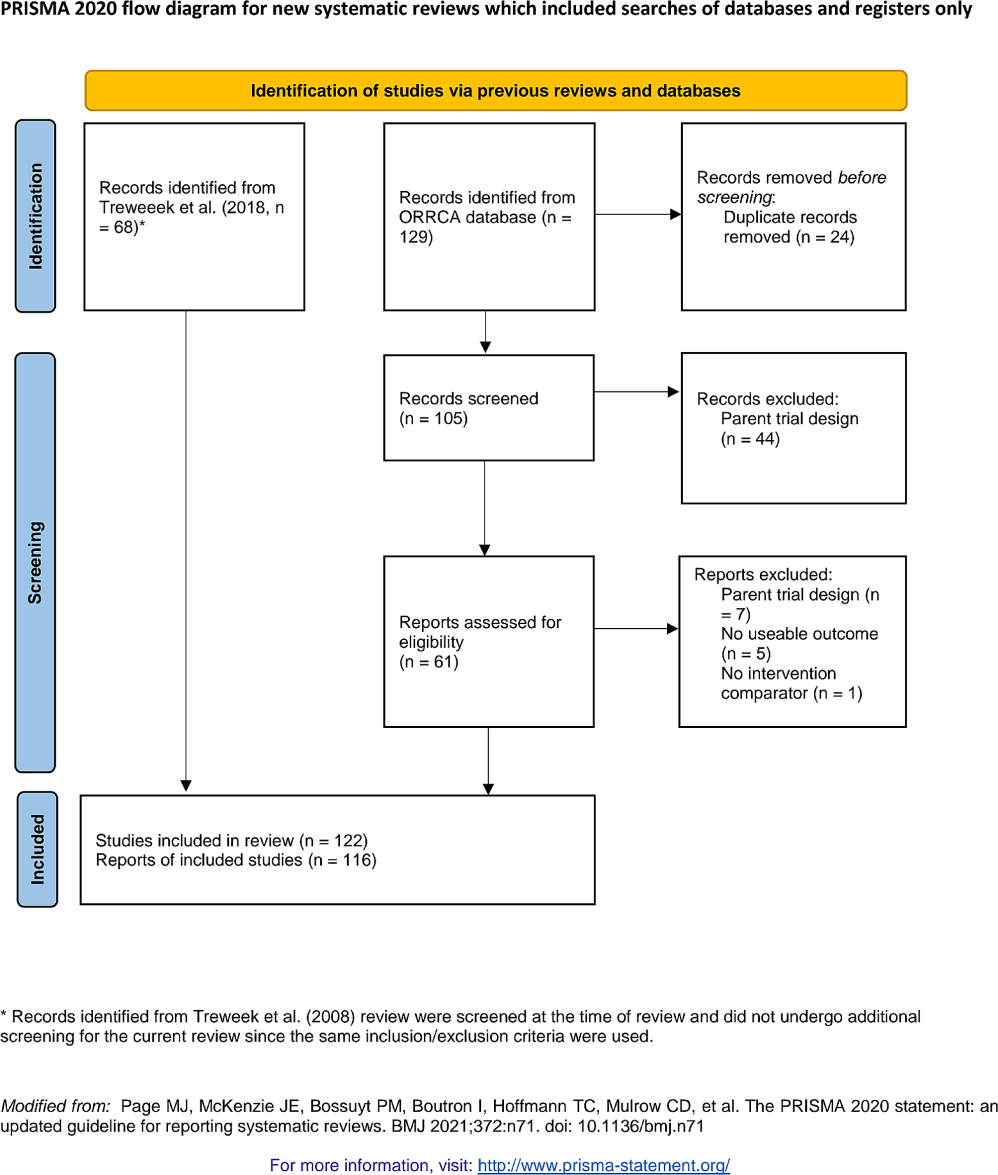
The PRISMA flow diagram for the review detailing the source of publications, number of abstracts and full texts screened, and number of publications included

Figure 1 outlines our PRISMA diagram for study identification and inclusion. The 68 papers from Treweek et al. (2018) were automatically included as they met all inclusion criteria [8]. The ORRCA database searches resulted in 129 additional records. After duplicates were removed (*n* = 24), we had 105 records to review for inclusion. A further 51 studies were deemed ineligible based on the wrong host trial design (e.g. survey study, non-randomized trial), 5 studies had no useable recruitment outcomes, and one had no comparator group. The remaining 48 studies identified from the ORRCA database searches were added to the Treweek et al. [8] review studies, for a total sample of 116 papers (see Additional file 1, Appendix B) [11, 21–135]. Five papers reported results from multiple recruitment intervention trials; therefore, we extracted data from 122 individual studies within the included publications.

The final sample of included recruitment studies is described in Table 1. Over half (64%) were published between 2010 and 2020. Most were conducted in the USA (43%), UK (33%), or Australia (9%). The majority (64%) asked potential participants to consider participation in a real trial, rather than a hypothetical trial (36%). The largest proportion (30%) focused on oncology trials. Trials were most frequently categorized as Phase III (41%), while many others reported recruitment to multiple trials that varied in phase (19%), and one third (34%) of studies could not be coded into a trial phase because of a lack of detail in describing the host trial or not fitting into the phase categories used (e.g., screening trials, supplement use). Approximately half of included studies reported information on mean/median participant age (*n* = 65), which ranged from 14.2 to 77.7 years. Approximately two thirds (*n* = 81) reported about participant gender (mean percent female = 62%), with most studies including both males and females (*n* = 62), while others only included females (*n* = 17) or males (*n* = 2).

**Table 1:** Descriptive characteristics of included studies (*n* = 122)

	Frequency (%)
Year of publication	
<= 1989	1 (0.8)
1990 to 1999	11 (9.0)
2000 to 2009	32 (26.2)
2010 to 2020	78 (63.9)
Country	
USA	53 (43.4)
UK	40 (32.8)
Australia	11 (9.0)
Canada	6 (4.9)
Other	12 (9.8)
Trial participation decision	
Real	78 (63.9)
Hypothetical	44 (36.1)
Clinical specialty of host trial	
Oncology	36 (29.5)
Cardiology	9 (7.4)
Injury prevention	8 (6.6)
Endocrinology	6 (4.9)
Psychiatry	5 (4.1)
Smoking cessation	5 (4.1)
Gynecology	5 (4.1)
Infectious disease	4 (3.3)
Orthopedics	4 (3.3)
Pulmonology	4 (3.3)
Neurology	3 (2.5)
Multiple specialties	9 (7.4)
Other	24 (19.7)
Host trial phase	
Phase II	4 (3.3)
Phase III	50 (41.0)
Phase IV	3 (2.5)
Multiple trials/phases	23 (18.9)
Other	42 (34.4)

**TIDieR reporting.** The frequency with which we were able to extract information relevant to each of the 12 TIDieR items for each study is presented in italics in Table 2. We identified many items as present for all studies: name/description, rationale, materials, procedure, and mode of delivery. Other items were reported with high frequency: provider (83%), location (94%), and intervention frequency (99%); while duration was less frequently reported (26%). The final four TIDieR items were rarely reported. These included tailoring (8%), modifications (0%), planned fidelity (5%), and actual fidelity (14%).

**Table 2:** Proportion of studies reporting information relevant to the 12 TIDieR checklist items and item-specific intervention details (*n* = 122)

Original TIDieR element	Descriptive item	Frequency (% of studies) for which descriptive item was extractable
1. Name/description	*Description of intervention*	*122 (100.0)*
Example:	“The CI [coaching intervention] was to provide flexible, individualized, nondirective basic education and support for patients in order to create a context of trust that promoted clinical trial enrollment.” [73]	
2. Why (rationale)	*Intervention rationale information*	*122 (100.0)*
Item details:	Rationale linked to intervention:	
	Rationale linked	68 (55.7)
	No clear link	38 (31.1)
	Not applicable	16 (13.1)
3. What (materials)	*Materials information*	*122 (100.0)*
Item details:	Materials used:	
	Modified recruitment document	41 (33.6)
	None	16 (13.1)
	Additional recruitment document	15 (12.3)
	Video	15 (12.3)
	Computer program/site	11 (9.0)
	Recruiter materials	9 (7.4)
	Other (e.g., incentives, SMS messages)	15 (12.3)
Item details:	Materials access provided:	
	No/partial materials access	80 (65.6)
	Access to full materials	31 (25.4)
	Not applicable	11 (9.0)
4. What (procedure)	*Intervention design information*	*122 (100.0)*
Item details:	Intervention design, ORRCA categories:	
	Modified information	67 (54.9)
	Modification to consent process	15 (12.3)
	Host trial design	13 (10.7)
	Changes during trial	10 (8.2)
	Recruiter/site intervention	10 (8.2)
	Incentives	5 (4.1)
	Other	2 (1.6)
5. Who (provider)	*Intervention provider*	*101 (82.8)*
Item details:	Type of intervention provider:	
	Member of research team	72 (59.0)
	Member of clinical care team	20 (16.4)
	Other	9 (7.4)
	Not reported	21 (17.2)
6. How (modes of delivery)	*Mode(s) of delivery*	*122 (100.0)*
Item details:	Primary mode(s) of delivery:*	
	Informational – Printed material	57 (46.7)
	Informational – Electronic	44 (36.1)
	Informational – Human Interactional	29 (23.8)
	Informational – Audio/visual	9 (7.4)
	Environmental change	3 (4.5)
	Other	6 (4.9)
7. Where (intervention location)	*Intervention location*	*115 (94.3)*
Item details:	Location type:	
	Hospital, clinic	50 (41.0)
	Non-clinical setting	39 (32.0)
	Multiple locations	7 (5.7)
	Virtual	19 (15.6)
	Not reported	7 (5.7)
8a. Frequency of intervention	*Information about frequency*	*121 (99.2)*
Item details:	Intervention frequency:	
	One-time event	110 (90.2)
	Multiple intervention times	11 (9.0)
	Not reported	1 (0.8)
8b. Duration of intervention	*Information about duration*	*32 (26.2)*
Item details:	Range in reported intervention duration (minutes)	5–774
9. Tailoring	*Information about tailoring*	*10 (8.2)*
Examples:	“Emails to the clinical sites from the central trial coordinators generally contained highly-tailored site-specific information about recruitment performance relative to goals,…” [32]	
	“The coach provided flexible social support and education addressing (1) general issues in the patient’s life (to establish rapport and show interest in the patient),… and (4) promotion of participation in clinical trials.” [73]	
10. Modifications during study	*Information about modifications*	*0 (0.0)*
11. Planned fidelity/adherence	*Information about planned fidelity*	*6 (4.9)*
Examples:	“The project coordinator reviewed records weekly to assure protocol adherence.” [37]	
	“The packs were placed in order of the random allocation list and then numbered sequentially by the researcher before being sent to the practice. By numbering the packs, the researcher had a record of which PIL type was in each pack, which enabled the researcher to monitor if packs were sent out in the correct randomized order.” [69]	
12. Actual fidelity/adherence	*Information about actual fidelity*	*17 (13.9)*
Examples:	“A few participants could not complete some of the tasks (17%, 14/89) due to technical or other problems.” [92]	
	“We reported higher reminder delivery in the SMS group (88% delivered) compared with the phone group (67% delivered, 78% if voicemail messages are also included). However, this apparent difference in intervention delivery fidelity is likely to be an artifact of how delivery was measured in each group.” [105]	

*Mode of delivery percentages add to more than 100% due to some interventions using multiple modes of delivery

**TIDieR descriptive items.** Results detailing the descriptive items based on TIDieR are summarized in Table 2. About half of studies (56%) demonstrated a clear link between the rationale and selected intervention, for example, “The rationale is that senior investigators would have better clinical judgment with which to assess study eligibility. Another common belief is that they exude an aura of expertise that might encourage wavering prospective subjects to participate.” [41]; and 31% did not demonstrate a clear link, for example, “We hypothesized that patients randomized to telephone-based follow-up would be more likely to attend for eligibility screening and be enrolled into the SCOPE trial than those randomized to mail based follow-up” [26]. A smaller portion (13%) did not state recruitment as a primary goal of the intervention (e.g., aim was to improve patient understanding), but did report on recruitment outcomes and was therefore rated as ‘not applicable’ for this item. Materials often amounted to informational documents that were either modified consent documents (34%) or documents in addition to standard consent documents (13%); less frequently, materials involved videos (12%) or computer programs/websites (9%). Authors provided access to full materials for only 25% of studies. The most common ORRCA category for intervention type was modified information presented to potential participants (55%). The intervention provider was most commonly a member of the research team (59%). The modes of delivery (from Marques et al. 2020 [19] ontology) were overwhelmingly informational, often in the form of printed (47%) or electronic (36%) materials. Only three studies used changes to the environment via electronic data capture systems (5%). While the modes of delivery could be captured by a single mode for most studies (n = 92, 75%) other more complex interventions required the selection of two (n = 26, 21%) or three (n = 4, 3%) modes to accurately describe the modes of delivery.

Intervention location was primarily in clinical settings (41%) such as hospitals or primary care clinics, but also frequently non-clinical settings (32%) such as universities, churches, and community centers. Interventions were most commonly administered at a single time point (90%), while others were conducted over 3 or more sessions/time points (9%; e.g., reminders, multiple informational sessions). When reported, the length of time recipients received the intervention ranged from as short as 5 min up to 13 h for multi-day informational sessions. Ten studies (8%) indicated interventions were tailored to participants in some way, such as audio taping the recruitment session for participants to take home, emails with site specific information for recruiters, and being assigned educational videos based on their trial knowledge or attitude scores from questionnaires (see Table 2 for example quotes). No studies reported any intervention modifications during the study. While six studies (5%) appeared to report plans to assess fidelity/adherence to the interventions, 14% (*n* = 17) reported actual fidelity/adherence observed during the study; whether it was that interventions were delivered as planned, or reporting minor issues in delivery, such as technical errors or site investigator non-compliance. We found fidelity reporting to be more detailed in some studies than others (see Table 2 for example quotes).

**Fig. 2:**
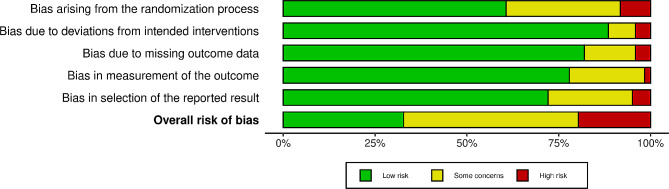
Summary of risk of bias ratings by domain and overall across studies (*n* = 122) [136]

**Risk of bias.** Risk of bias ratings were distributed across the low risk (*n* = 40, 33%), some concerns (*n* = 57, 47%), and high risk (*n* = 25, 21%) categories. Figure 2 presents a summary of the ratings by domain. Bias arising from the randomization process was the biggest source of potential bias in the included intervention studies, while the other four domains were often rated as a low source of potential bias.

## Discussion

Strategies designed to improve clinical trial recruitment are typically not conceptualized as complex interventions, despite the fact that they often have many of the defining characteristics of complex interventions [10] such as multiple components, varying groups/individuals targeted by the interventions (e.g., potential participants, recruiters, health care providers), and levels of flexibility of tailoring involved. In response to numerous calls for clarity around understanding and reporting of important aspects of trial recruitment [137–139], we sought to use the TIDieR checklist as a guide to describe the reporting of trial recruitment intervention studies. Our findings suggest that TIDieR can be used to provide a useful description of the main components of complex trial recruitment interventions, highlights variability in the reporting of these interventions, and suggests ways in which reporting of these interventions can be improved.

Our first aim was to understand to what extent we could identify/extract information about each of the 12 items within recruitment intervention studies. While the framework has been applied to a variety of health service interventions [140], it is new to the discussion of recruitment interventions, perhaps because such interventions often focus on simple outcomes (e.g., trial enrolment). However, recruitment interventions are often complex in other aspects, such as number of components and variety of individuals targeted by the intervention [10]. While many items were reported with relatively high consistency for all studies, the last four items were very rarely reported. Although tailoring and modifications would only be reported when present, they were present for surprisingly few studies considering they may be important components of complex interventions. It was unclear whether a lack of evidence of these two items was due to poor reporting or irrelevant to the intervention. Perhaps TIDieR guidance should include recommending statements when these items are not present in order for readers to better understand all intervention components, whether used or not. Recruitment interventions are unlikely to be delivered with 100% fidelity, which may affect the associated recruitment outcomes. However, very few studies reported on fidelity or lack thereof, and even fewer studies provided evidence of a pre-specified fidelity plan. It is unclear whether any lack of effect for these complex interventions are due to the interventions themselves or a problem with intervention delivery. It appears that despite meeting many criteria for complex interventions, recruitment interventions are not being considered as such, reflected by the brevity and simplicity with which they are currently being reported. Trialists should seek to measure and report these details of their interventions with increased detail and consistency, particularly around fidelity and tailoring/modification, in order to advance the literature in this area and allow for more rigorous evaluation across studies.

Insights can be drawn from the items reported with high frequency as well. Materials, procedure, modes of delivery, and frequency, were identified as present for almost every study, indicating these items tend to be well reported in the literature; this may reflect a general belief that these are the most important components of recruitment interventions. It is unclear what level of detail is optimal for assessing the most effective intervention components across studies. Finally, despite extensive piloting, decisions around the reporting of two items (provider, location) often required extensive discussion between raters before consensus could be achieved, suggesting that a more detailed understanding of how these constructs manifest themselves in recruitment strategies and studies is needed. Perhaps a more systematic approach is needed in order to ensure more consistency in reporting all TIDieR items in a way that readers can understand and replicate when appropriate. Methods such as those used in Cochrane reviews, with rigorous consensus processes and standardized extraction tools may apply well to recruitment interventions studies to improve the reporting of intervention details in a way that facilitates replication by future trialists.

Our second aim was to use TIDieR as a guide to describe useful variation among recruitment intervention studies. Our work shows that the TIDieR checklist can be used as a guiding framework for describing important elements of recruitment strategies from a diverse set of recruitment intervention studies, spanning many clinical domains, countries of conduct, and diverse nature of the interventions themselves. Some items provided more useful descriptive information than others; although an intervention name/description was present for all studies, inclusion in TIDieR may not provide much value in reporting relevant intervention details since it could be considered a brief summary of the other more specific TIDieR items. This may also explain why we were unable to explore this item in more detail by developing descriptive categories.

Our approach also allows for combination with other descriptive frameworks that provide more detail on TIDieR-inspired domains. We employed ORRCA intervention design categories [14] to detail the TIDieR procedure domain as it captured the diverse nature of recruitment intervention procedures in a way that highlighted the ‘active ingredients’ of these types of interventions. We also found that using the Marques et al. [19] ontology to categorize modes of delivery highlighted that most of these interventions have thus far focused primarily on various informational modes rather than environmental (e.g., material incentives and reminders) or somatic (e.g., physical stimuli such as light or temperature). The ontology did not capture instances where the intervention was a change to parent trial design (e.g., Zelen design, removing control groups) unless participants were explicitly informed about the trial design (informational mode of delivery). This suggests there may be a benefit to identifying other frameworks beyond Marques et al. to describe more thoroughly the range of modes of delivery used in recruitment interventions.

Trialists may use recruitment interventions in clinical trials from a wide range of clinical specialties, patient populations, and trial phases. We found the detail with which authors described host trials varied across studies. As many as one third were so briefly described that a trial phase could not be determined and was not reported. The motivations to participate in a phase I trial that is testing the safety of a new and experimental drug may be different from motivation to participate in a phase III trial that is testing treatment efficacy against other similarly effective drugs. Therefore, understanding the trial phase and how the findings of a recruitment intervention that is successful in one setting translates (or not) to another is critical. For example, if an intervention proves to be effective in multiple studies across a range of phase III host trials, we cannot assume that it will be effective once implemented in a phase I trial; and therefore warrants further development and evaluation. Consideration should be given as to what host trial details should be reported with greater consistency and clarity when reporting on trails within a trial.

### Limitations

Our study had four main limitations. First, we were unable to contact authors of included studies for missing information. While this may have affected risk of bias ratings regarding the reporting of results, we attempted to correct for this by leniency in reporting pre-specified analysis plans since their presence would not have a big impact on reporting of recruitment outcome numbers or rates. Second, due to time and resource limitations, we were not able to search multiple databases for eligible publications; therefore, we may have missed some relevant studies. However, the review and database from which we collected publications both used a systematic approach to searching and screening and we are confident that the vast majority of relevant studies have been included. Third, we elected not to perform meta-analyses on the included papers to explore whether specific TIDieR items or characteristics of items related to recruitment effect sizes. Considering the heterogeneity of included studies, combined with the finding that many studies were rated as high or some concerns for potential bias, we would have relatively low power to detect meaningful differences in effect sizes. Finally, we were not able to operationalize all TIDieR items into descriptive categorical variables; more work needs to be done to specify how these items can be assessed to facilitate better reporting in the recruitment intervention literature in the future.

### Future directions

Future work should involve testing the methods used here to develop descriptive extraction variables based on TIDieR items with other intervention types in other reviews to gain a better understanding of whether the categories and methods used here also apply to other settings or whether further development of each item is needed. In addition, further development of the items collected as quotations would aid in more accurately describing and evaluating these interventions.

There may be other key characteristics of recruitment interventions not captured by the TIDieR checklist or the modifications we made in the current study that warrant consideration. Future research should explore the utility of additional reporting items for recruitment interventions, such as adaptability of the intervention to other trial settings or the intervention target (e.g., potential participants, trial recruiters), in addition to who is delivering the intervention [141]. Recent research has also focused on the carbon footprint of clinical trials [142–144]. Recruitment interventions may have a direct impact on the carbon footprint of the trials in which they are embedded (e.g., minimizing study materials, speeding up recruitment, implementing virtual trial visits [142]). Including the potential environmental impact as a TIDieR item may help assess the potential longevity of specific recruitment practices and other interventions when deciding which strategies are most appropriate for the trials in question.

It may also be worth exploring other frameworks to see if they might compliment or prove superior to the methods used here in describing the important components of recruitment interventions. Other ontologies, similar to the mode of delivery ontology developed by Marques et al. [19], are currently being developed to further explore the details of other TIDieR elements in greater detail [145, 146]. These ontologies may provide the structure and detail needed to better describe and understand the components of recruitment interventions. This study is part of a larger review examining other factors that may relate to recruitment intervention effectiveness. For example, exploring the use of behavior change techniques in recruitment interventions may provide further insight into what components are most effective in improving recruitment outcomes. Also, while theory use is included as part of TIDieR item 2, we chose to focus the current evaluation on intervention rationale to reduce duplication of work. We will explore whether and how these studies use theory in selecting, developing, and implementing recruitment interventions in detail elsewhere. We will also evaluate the inclusion of participant-centered involvement in these interventions and how these methods differs across studies.

Once a more detailed and comprehensive set of variables is developed, and with the continually growing body of recruitment intervention studies, future research should examine whether these elements can help identify the features most predictive of effective recruitment interventions and in what settings through meta-analyses. This in turn could lead to more efficient trials, thereby reducing costs and bringing new treatments and innovations to patients faster.

## Conclusions

We extracted recruitment intervention details and information about reporting from a large, diverse sample of 122 studies evaluating these interventions in randomized and quasi-randomized trials using the TIDieR checklist as a guide. We were able to extract relevant intervention details on important elements of these interventions using a mix of categorical variables and quotations, indicating the TIDieR checklist items fit reasonably well to recruitment interventions. We found that these key components were variably described across studies, with some items being reported more consistently and clearly than others, highlighting areas in which reporting could be improved to facilitate accumulation of knowledge around recruitment practices. Our operationalisations of TIDieR descriptive intervention details were a first-draft attempt to characterize recruitment practices systematically; future research should explore the benefit of additional items (e.g. intervention targets, carbon footprints) or frameworks to improve description of these interventions, and evaluate which components are most related to improved recruitment outcomes. The current findings provide an initial template by which trialists can conceptualise their recruitment efforts as complex interventions for which planning and optimization guidance already exists.

### Electronic supplementary material

Below is the link to the electronic supplementary material.

Supplementary Material 1

Supplementary Material 2

## Data Availability

All data generated or analysed during this study are included in this published article as Additional file 2.

## Abbreviations

ORRCA: Online Resource for Research in Clinical triAls
PICOS: Participants, interventions, comparator, outcomes, study designs
PRISMA: Preferred Reporting Items for Systematic Reviews and Meta-Analyses
RoB: Risk of bias
TIDieR: Template for Intervention Description and Replication
