# Supplementary material for: Describing the content of trial recruitment interventions using the TIDieR reporting checklist: a systematic methodology review
Source: BMC Med Res Methodol. 2024 Apr 8;24:85. doi: 10.1186/s12874-024-02195-5 (PMC11000410; doi:10.1186/s12874-024-02195-5)
Supplement: Supplementary file 1 — Supplementary Material 1 [file 12874_2024_2195_MOESM1_ESM.docx]

|  | **Item #** | **Checklist item** | **Location where item is reported** |
| --- | --- | --- | --- |
| **TITLE** | | |  |
| Title | 1 | Identify the report as a systematic review. | Page 1 |
| **ABSTRACT** | | |  |
| Abstract | 2 | See the PRISMA 2020 for Abstracts checklist. | Pages 1-2 |
| **INTRODUCTION** | | |  |
| Rationale | 3 | Describe the rationale for the review in the context of existing knowledge. | Page 2 |
| Objectives | 4 | Provide an explicit statement of the objective(s) or question(s) the review addresses. | Page 2 |
| **METHODS** | | |  |
| Eligibility criteria | 5 | Specify the inclusion and exclusion criteria for the review and how studies were grouped for the syntheses. | Page 3 |
| Information sources | 6 | Specify all databases, registers, websites, organisations, reference lists and other sources searched or consulted to identify studies. Specify the date when each source was last searched or consulted. | Page 3 |
| Search strategy | 7 | Present the full search strategies for all databases, registers and websites, including any filters and limits used. | Pages 2-3 |
| Selection process | 8 | Specify the methods used to decide whether a study met the inclusion criteria of the review, including how many reviewers screened each record and each report retrieved, whether they worked independently, and if applicable, details of automation tools used in the process. | Page 3 |
| Data collection process | 9 | Specify the methods used to collect data from reports, including how many reviewers collected data from each report, whether they worked independently, any processes for obtaining or confirming data from study investigators, and if applicable, details of automation tools used in the process. | Page 3 |
| Data items | 10a | List and define all outcomes for which data were sought. Specify whether all results that were compatible with each outcome domain in each study were sought (e.g. for all measures, time points, analyses), and if not, the methods used to decide which results to collect. | n/a |
|  | 10b | List and define all other variables for which data were sought (e.g. participant and intervention characteristics, funding sources). Describe any assumptions made about any missing or unclear information. | Pages 3-5 |
| Study risk of bias assessment | 11 | Specify the methods used to assess risk of bias in the included studies, including details of the tool(s) used, how many reviewers assessed each study and whether they worked independently, and if applicable, details of automation tools used in the process. | Page 5 |
| Effect measures | 12 | Specify for each outcome the effect measure(s) (e.g. risk ratio, mean difference) used in the synthesis or presentation of results. | n/a |
| Synthesis methods | 13a | Describe the processes used to decide which studies were eligible for each synthesis (e.g. tabulating the study intervention characteristics and comparing against the planned groups for each synthesis (item #5)). | n/a |
|  | 13b | Describe any methods required to prepare the data for presentation or synthesis, such as handling of missing summary statistics, or data conversions. | Page 5 |
|  | 13c | Describe any methods used to tabulate or visually display results of individual studies and syntheses. | Page 5 |
|  | 13d | Describe any methods used to synthesize results and provide a rationale for the choice(s). If meta-analysis was performed, describe the model(s), method(s) to identify the presence and extent of statistical heterogeneity, and software package(s) used. | Page 5 |
|  | 13e | Describe any methods used to explore possible causes of heterogeneity among study results (e.g. subgroup analysis, meta-regression). | n/a |
|  | 13f | Describe any sensitivity analyses conducted to assess robustness of the synthesized results. | n/a |
| Reporting bias assessment | 14 | Describe any methods used to assess risk of bias due to missing results in a synthesis (arising from reporting biases). | n/a |
| Certainty assessment | 15 | Describe any methods used to assess certainty (or confidence) in the body of evidence for an outcome. | n/a |
| **RESULTS** | | |  |
| Study selection | 16a | Describe the results of the search and selection process, from the number of records identified in the search to the number of studies included in the review, ideally using a flow diagram. | Page 5; Figure 1 |
|  | 16b | Cite studies that might appear to meet the inclusion criteria, but which were excluded, and explain why they were excluded. | Pages 5 |
| Study characteristics | 17 | Cite each included study and present its characteristics. | Page 5; Table 1 |
| Risk of bias in studies | 18 | Present assessments of risk of bias for each included study. | Pages 7, 9; Figure 2 |
| Results of individual studies | 19 | For all outcomes, present, for each study: (a) summary statistics for each group (where appropriate) and (b) an effect estimate and its precision (e.g. confidence/credible interval), ideally using structured tables or plots. | n/a |
| Results of syntheses | 20a | For each synthesis, briefly summarise the characteristics and risk of bias among contributing studies. | Pages 5-9; Figure 2 |
|  | 20b | Present results of all statistical syntheses conducted. If meta-analysis was done, present for each the summary estimate and its precision (e.g. confidence/credible interval) and measures of statistical heterogeneity. If comparing groups, describe the direction of the effect. | Pages 5-9; Tables 1-2 |
|  | 20c | Present results of all investigations of possible causes of heterogeneity among study results. | n/a |
|  | 20d | Present results of all sensitivity analyses conducted to assess the robustness of the synthesized results. | n/a |
| Reporting biases | 21 | Present assessments of risk of bias due to missing results (arising from reporting biases) for each synthesis assessed. | n/a |
| Certainty of evidence | 22 | Present assessments of certainty (or confidence) in the body of evidence for each outcome assessed. | n/a |
| **DISCUSSION** | | |  |
| Discussion | 23a | Provide a general interpretation of the results in the context of other evidence. | Pages 9-12 |
|  | 23b | Discuss any limitations of the evidence included in the review. | Page 11 |
|  | 23c | Discuss any limitations of the review processes used. | Page 11 |
|  | 23d | Discuss implications of the results for practice, policy, and future research. | Pages 11-12 |
| **OTHER INFORMATION** | | |  |
| Registration and protocol | 24a | Provide registration information for the review, including register name and registration number, or state that the review was not registered. | n/a |
|  | 24b | Indicate where the review protocol can be accessed, or state that a protocol was not prepared. | n/a |
|  | 24c | Describe and explain any amendments to information provided at registration or in the protocol. | n/a |
| Support | 25 | Describe sources of financial or non-financial support for the review, and the role of the funders or sponsors in the review. | Page 12 |
| Competing interests | 26 | Declare any competing interests of review authors. | Page 12 |
| Availability of data, code and other materials | 27 | Report which of the following are publicly available and where they can be found: template data collection forms; data extracted from included studies; data used for all analyses; analytic code; any other materials used in the review. | Page 12 |

*From:*  Page MJ, McKenzie JE, Bossuyt PM, Boutron I, Hoffmann TC, Mulrow CD, et al. The PRISMA 2020 statement: an updated guideline for reporting systematic reviews. BMJ 2021;372:n71. doi: 10.1136/bmj.n71

For more information, visit: <http://www.prisma-statement.org/>

**Appendix B - List of publications included in the review**

Abd-Elsayed AA, Sessler DI, Mendoza-Cuartas M, Dalton JE, Said T, Meinert J, et al. A randomized controlled study to assess patients’ understanding of and consenting for clinical trials using two different consent form presentations. Minerva Anestesiol. 2012;78(5):564-73.

Abhyankar P, Bekker HL, Summers BA, Velikova G. Why values elicitation techniques enable people to make informed decisions about cancer trial participation. Health Expect. 2011;14 Suppl 1:20-32.

Annett RD, Brody JL, Scherer DG, Turner CW, Dalen J, Raissy H. A randomized study of a method for optimizing adolescent assent to biomedical research. AJOB Empir Bioeth. 2017;8(3):189-97.

Arundel C, Jefferson L, Bailey M, Cockayne S, Hicks K, Loughrey L, et al. A randomized, embedded trial of pre-notification of trial participation did not increase recruitment rates to a falls prevention trial. J Eval Clin Pract. 2017;23(1):73-8.

Avenell A, Grant AM, McGeeb M, McPherson G, Campbell MK, McGee MAftRTMG. The effects of an open design on trial participant recruitment, compliance and retention - a randomized controlled trial comparison with a blinded, placebo-controlled design. Clinical Trials. 2004;1:490-8.

Bentley JP, Thacker PG. The influence of risk and monetary payment on the research participation decision making process. J Med Ethics. 2004;30(3):293-8.

Bergenmar M, Johansson H, Wilking N, Hatschek T, Brandberg Y. Audio-recorded information to patients considering participation in cancer clinical trials - a randomized study. Acta Oncol. 2014;53(9):1197-204.

Bickmore TW, Utami D, Matsuyama R, Paasche-Orlow MK. Improving access to online health information with conversational agents: A randomized controlled experiment. J Med Internet Res. 2016;18(1):e1.

Bobb MR, Van Heukelom PG, Faine BA, Ahmed A, Messerly JT, Bell G, et al. Telemedicine provides noninferior research informed consent for remote study enrollment: A randomized controlled trial. Acad Emerg Med. 2016;23(7):759-65.

Bracken K, Keech A, Hague W, Kirby A, Robledo KP, Allan C, et al. Telephone call reminders did not increase screening uptake more than SMS reminders: a recruitment study within a trial. J Clin Epidemiol. 2019;112:45-52.

Brierley G, Richardson R, Torgerson DJ. Using short information leaflets as recruitment tools did not improve recruitment: a randomized controlled trial. J Clin Epidemiol. 2012;65(2):147-54.

Brown SD, Partee PN, Feng J, Quesenberry CP, Hedderson MM, Ehrlich SF, et al. Outreach to diversify clinical trial participation: A randomized recruitment study. Clin Trials. 2015;12(3):205-11.

Chen F, Rahimi K, Haynes R, Naessens K, Taylor-Clarke M, Murray C, et al. Investigating strategies to improve attendance at screening visits in a randomized trial. Trials. 2011;12(Suppl 1).

Cockayne S, Fairhurst C, Adamson J, Hewitt C, Hull R, Hicks K, et al. An optimised patient information sheet did not significantly increase recruitment or retention in a falls prevention study: an embedded randomised recruitment trial. Trials. 2017;18(1):144.

Cooper KG, Grant AM, Garratt AM. The impact of using a partially randomised patient preference design when evaluating alternative managements for heavy menstrual bleeding. British Journal of Obstetrics and Gynaecology. 1997;104:1367-73.

Coyne C, Xu R, Raich P, Plomer K, Dignan M, Wenzel L, et al. Randomized, controlled trial of an easy-to-read informed consent statement for clinical trial participation: a study of the Eastern Cooperative Oncology Group. . Journal of Clinical Oncology 2003;21(5):836–42.

Crane MM, LaRose JG, Espeland MA, Wing RR, Tate DF. Recruitment of young adults for weight gain prevention: randomized comparison of direct mail strategies. Trials. 2016;17(1):282.

Dear RF, Barratt AL, Askie LM, Butow PN, McGeechan K, Crossing S, et al. Impact of a cancer clinical trials web site on discussions about trial participation: a cluster randomized trial. Ann Oncol. 2012;23(7):1912-8.

DiGuiseppi C, Goss C, Xu S, Magid D, Graham A. Telephone screening for hazardous drinking among injured patients seen in acute care clinics: feasibility study. Alcohol Alcohol. 2006;41(4):438-45.

Du W, Mood D, Gadgeel S, Simon MS. An educational video to increase clinical trials enrollment among breast cancer patients. Breast Cancer Res Treat. 2009;117(2):339-47.

Du W, Mood D, Gadgeel S, Simon MS. An educational video to increase clinical trials enrollment among lung cancer patients. J Thorac Oncol. 2008;3:23–9.

Ellis PM, Butow PN, Tattersall MH. Informing breast cancer patients about clinical trials: a randomized clinical trial of an educational booklet. Ann Oncol. 2002;13(9):1414-23.

Ethier JF, Curcin V, McGilchrist MM, Choi Keung SNL, Zhao L, Andreasson A, et al. eSource for clinical trials: Implementation and evaluation of a standards-based approach in a real world trial. Int J Med Inform. 2017;106:17-24.

Felicitas-Perkins JQ, Palalay MP, Cuaresma C, Ho RCS, Chen Jr. MS, Dang J, et al. A pilot study to determine the effect of an educational DVD in Philippine languages on cancer clinical trial participation among Filipinos in Hawai‘i. Hawai'i Journal of Medicine & Public Health. 2017;76(7):171-7.

Fleissig A, Jenkins V, Fallowfield L. Results of an intervention study to improve communication about randomised clinical trials of cancer therapy. European Journal of Cancer. 2001;37:322-31.

Ford ME, Havstad SL, Davis SD. A randomized trial of recruitment methods for older African American men in the Prostate, Lung, Colorectal and Ovarian (PLCO) cancer screening trial. Clinical Trials. 2004;1:343-51.

Foss KT, Kjaergaard J, Stensballe LG, Greisen G. Recruiting to clinical trials on the telephone - a randomized controlled trial. Trials. 2016;17(1):552.

Fowell A, Johnstone R, Finlay I, Russell D, Russell I. Design of trials with dying patients: a feasibility study of cluster randomisation versus randomised consent. Palliative Medicine. 2006;20:799-804.

Fracasso PM, Goodner SA, Creekmore AN, Morgan HP, Foster DM, Hardmon AA, et al. Coaching intervention as a strategy for minority recruitment to cancer clinical trials. Journal of Oncology Practice. 2013;9(6):294-9.

Free C, Hoile E, Robertson S, Knight R. Three controlled trials of interventions to increase recruitment to a randomized controlled trial of mobile phone based smoking cessation support. Clinical Trials 2010;7:265–73.

Free CJ, Hoile E, Knight R, Robertson S, Devries KM. Do messages of scarcity increase trial recruitment? Contemp Clin Trials. 2011;32(1):36-9.

Freer Y, McIntosh N, Teunisse S, Anand KJ, Boyle EM. More information, less understanding: a randomized study on consent issues in neonatal research. Pediatrics. 2009;123(5):1301-5.

Frew PM, Omer SB, Parker K, Bolton M, Schamel J, Shapiro E, et al. Delivering a "dose of hope": a faith-based program to increase older african americans' participation in clinical trials. JMIR Res Protoc. 2015;4(2):e64.

Fureman I, Meyers K, McLellan AT, Metzger D, Woody G. Evaluation of a video supplement to informed consent: Injection drug users and preventative HIV vaccine efficacy trials. AIDS Education and Prevention. 1997;9(4):330-41.

Garvelink MM, Freitas A, Menear M, Briere N, Stacey D, Legare F. In for a penny, in for a pound: the effect of pre-engaging healthcare organizations on their subsequent participation in trials. BMC Res Notes. 2015;8:751.

Graham A, Goss C, Xu S, Magid DJ, DiGuiseppi C. Effect of using different modes to administer the AUDIT-C on identification of hazardous drinking and acquiescence to trial participation among injured patients. Alcohol Alcohol. 2007;42(5):423-9.

Halpern SD, Karlawish JHT, Casarett D, Berlin JA, Asch DA. Empirical assessment of whether moderate payments are undue or unjust inducements for participation in clinical trials. Arch Intern Med. 2004;164:801-3.

Hemminki E, Hovi SL, Veerus P, Sevon T, Tuimala R, Rahu M, et al. Blinding decreased recruitment in a prevention trial of postmenopausal hormone therapy. J Clin Epidemiol. 2004;57(12):1237-43.

Hughes-Morley A, Hann M, Fraser C, Meade O, Lovell K, Young B, et al. The impact of advertising patient and public involvement on trial recruitment: embedded cluster randomised recruitment trial. Trials. 2016;17(1):586.

Hutchison C, Cowan C, McMahon T, Paul J. A randomised controlled study of an audiovisual patient information intervention on informed consent and recruitment to cancer clinical trials. Br J Cancer. 2007;97(6):705-11.

Ives N, Troop M, Waters A, Davies S, Higgs C, Easterbrook P. Does an HIV clinical trial information booklet improve patient knowledge and understanding of HIV clinical trials? HIV Medicine. 2001;2:241-9.

Jacobsen PB, Wells KJ, Meade CD, Quinn GP, Lee JH, Fulp WJ, et al. Effects of a brief multimedia psychoeducational intervention on the attitudes and interest of patients with cancer regarding clinical trial participation: a multicenter randomized controlled trial. J Clin Oncol. 2012;30(20):2516-21.

Jennings CG, MacDonald TM, Wei L, Brown MJ, McConnachie L, Mackenzie IS. Does offering an incentive payment improve recruitment to clinical trials and increase the proportion of socially deprived and elderly participants? Trials. 2015;16:80.

Jeste DV, Palmer BW, Golshan S, Eyler LT, Dunn LB, Meeks T, et al. Multimedia consent for research in people with schizophrenia and normal subjects: a randomized controlled trial. Schizophr Bull. 2009;35(4):719-29.

Karunaratne AS, Korenman SG, Thomas SL, Myles PS, Komesaroff PA. Improving communication when seeking informed consent: a randomised controlled study of a computer-based method for providing information to prospective clinical trial participants. MJA. 2010;192:388-92.

Kendrick D, Watson M, Dewey M, Woods AJ. Does sending a home safety questionnaire increase recruitment to an injury prevention trial? A randomised controlled trial. J Epidemiol Community Health. 2001;55:845–6.

Kerr CEP, Robinson EJ, Lilford RJ, Edwards SJL, Braunholtz DA, Stevens AJ. The impact of describing clinical trial treatments as new or standard. Patient Education and Counseling. 2004;53(1):107-13.

Kimmick GG, Peterson BL, Kornblith AB, Mandelblatt J, Johnson JL, Wheeler J, et al. Improving accrual of older persons to cancer treatment trials: a randomized trial comparing an educational intervention with standard information: CALGB 360001. J Clin Oncol. 2005;23(10):2201-7.

Larkey LK, Staten LK, Ritenbaugh C, Hall RA, Buller DB, Bassford T, et al. Recruitment of Hispanic women to the Women’s Health Initiative: the case of Embajadoras in Arizona. Controlled Clinical Trials 2002;23:289–98.

Lee H, Hubscher M, Moseley GL, Kamper SJ, Traeger AC, Skinner IW, et al. An embedded randomised controlled trial of a Teaser Campaign to optimise recruitment in primary care. Clin Trials. 2017;14(2):162-9.

Liénard J-L, Quinaux E, Fabre-Guillevin E, Piedbois P, Jouhaud A, Decoster G, et al. Impact of on-site initiation visits on patient recruitment and data quality in a randomized trial of adjuvant chemotherapy for breast cancer. Clinical Trials. 2006;3:486-92.

Litchfield J, Freeman J, Schou H, Elsley M, Fuller R, Chubb B. Is the future for clinical trials internet-based? A cluster randomized clinical trial. Clinical Trials. 2005;2:72-9.

Llewellyn-Thomas HA, McGreal MJ, Thiel EC. Cancer patients’ decision making and trial-entry preferences: The effects of "framing" information about short-term toxicity and long-term survival. Med Decis Making. 1995;15:4-12.

Llewellyn-Thomas HA, Thiel EC, Sem FWC, Harrison Woermke DE. Presenting clinical trial information: a comparison of methods. Patient Education and Counseling. 1995;25:97-107.

MacQueen KM, Chen M, Ramirez C, Nnko SE, Earp KM. Comparison of closed-ended, open-ended, and perceived informed consent comprehension measures for a mock HIV prevention trial among women in Tanzania. PLoS One. 2014;9(8):e105720.

Man MS, Healthlines Study G, Rick J, Bower P, Group M-S. Improving recruitment to a study of telehealth management for long-term conditions in primary care: two embedded, randomised controlled trials of optimised patient information materials. Trials. 2015;16:309.

Mandelblatt J, Kaufman E, Sheppard VB, Pomeroy J, Kavanaugh J, Canar J, et al. Breast cancer prevention in community clinics: will low-income Latina patients participate in clinical trials? Prev Med. 2005;40(6):611-8.

Maxwell AE, Parker RA, Drever J, Rudd A, Dennis MS, Weir CJ, et al. Promoting Recruitment using Information Management Efficiently (PRIME): a stepped-wedge, cluster randomised trial of a complex recruitment intervention embedded within the REstart or Stop Antithrombotics Randomised Trial. Trials. 2017;18(1):623.

Meropol NJ, Wong YN, Albrecht T, Manne S, Miller SM, Flamm AL, et al. Randomized trial of a web-based intervention to address barriers to clinical trials. J Clin Oncol. 2016;34(5):469-78.

Miller NL, Markowitz JC, Kocsis JH, Leon AC, Brisco ST, Garno JL. Cost effectiveness of screening for clinical trials by research assistants versus senior investigators. Journal of Psychiatric Research. 1999;33:81-5.

Monaghan H, Richens A, Colman S, Currie R, Girgis S, Jayne K, et al. A randomised trial of the effects of an additional communication strategy on recruitment into a large-scale, multi-centre trial. Contemp Clin Trials. 2007;28(1):1-5.

Mudano AS, Gary LC, Oliveira AL, Melton M, Wright NC, Curtis JR, et al. Using tablet computers compared to interactive voice response to improve subject recruitment in osteoporosis pragmatic clinical trials: feasibility, satisfaction, and sample size. Patient Prefer Adherence. 2013;7:517-23.

Myles PS, Fletcher HE, Cairo S, Madder H, McRae R, Cooper J, et al. Randomized trial of informed consent and recruitment for clinical trials in the immediate preoperative period. Anesthesiology. 1999;91:969-78.

Nystuen P, Hagen KB. Telephone reminders are effective in recruiting nonresponding patients to randomized controlled trials. J Clin Epidemiol. 2004;57(8):773-6.

Paris A, Deygas B, Cornu C, Thalamas C, Maison P, Duale C, et al. Improved informed consent documents for biomedical research do not increase patients' understanding but reduce enrolment: a study in real settings. Br J Clin Pharmacol. 2015;80(5):1010-20.

Parker A, Knapp P, Treweek S, Madhurasinghe V, Littleford R, Gallant S, et al. The effect of optimised patient information materials on recruitment in a lung cancer screening trial: an embedded randomised recruitment trial. Trials. 2018;19(1):503.

Parker C, Snyder R, Jefford M, Dilts D, Wolfe R, Millar J. A randomized controlled trial of an additional funding intervention to improve clinical trial enrollment. J Natl Compr Canc Netw. 2017;15(9):1104-10.

Paul C, Courtney R, Sanson-Fisher R, Carey M, Hill D, Simmons J, et al. A randomized controlled trial of the effectiveness of a pre-recruitment primer letter to increase participation in a study of colorectal screening and surveillance. BMC Med Res Methodol. 2014;14:44.

Paul J, Iveson T, Midgley R, Harkin A, Masterton M, Alexander L, et al. Choice of randomisation time-point in non-inferiority studies of reduced treatment duration: experience from the SCOT study. Trials. 2011;12(S1).

Perrone F, De Placido S, Giusti C, Gallo C. Looking for consent in RCTs: a randomised trial with surrogate patients [La richiesta del consenso nella ricerca clinica: uno studio randomizzato in soggetti sani]. Epidemiologia e Prevenzione. 1995;19:282–90.

Pighills A, Torgerson DJ, Sheldon T. Publicity does not increase recruitment to falls prevention trials: the results of two quasi-randomized trials. J Clin Epidemiol. 2009;62(12):1332-5.

Simel DL, Feussner JR. A randomized controlled trial comparing quantitative informed consent formats. J Clin Epidemiol. 1991;44(8):771-7.

Simes RJ, Tattersall MH, Coates AS, Raghaven D, Solomon HJ, Smartt H. Randomised comparison of procedures for obtaining informed consent in clinical trials of treatment for cancer. BMJ. 1986;293:1065-8.

Tehranisa JS, Meurer WJ. Can response-adaptive randomization increase participation in acute stroke trials? Stroke. 2014;45(7):2131-3.

Tilley BC, Mainous AG, 3rd, Elm JJ, Pickelsimer E, Soderstrom LH, Ford ME, et al. A randomized recruitment intervention trial in Parkinson's disease to increase participant diversity: early stopping for lack of efficacy. Clin Trials. 2012;9(2):188-97.

Treschan TA, Scheck T, Kober A, Fleischmann E, Birkenberg B, Petschnigg B, et al. The influence of protocol pain and risk on patients’ willingness to consent for clinical studies: A randomized trial. Anesth Analg. 2003;96:498–506.

Trevena L, Irwig L, Barratt A. Impact of privacy legislation on the number and characteristics of people who are recruited for research: a randomised controlled trial. J Med Ethics. 2006;32(8):473-7.

Treweek S, Barnett K, Maclennan G, Bonetti D, Eccles MP, Francis JJ, et al. E-mail invitations to general practitioners were as effective as postal invitations and were more efficient. J Clin Epidemiol. 2012;65(7):793-7.

Veerus P, Fischer K, Hemminki E, Hovi SL, Hakama M. Effect of characteristics of women on attendance in blind and non-blind randomised trials: analysis of recruitment data from the EPHT Trial. BMJ Open. 2016;6(10):e011099.

Wadland WC, Hughes JR, Secker-Walker RH, Bronson DL, Fenwick J. Recruitment in a primary care trial on smoking cessation. Fam Med. 1990;22:201-4.

Weinfurt KP, Hall MA, Dinan MA, DePuy V, Friedman JY, Allsbrook JS, et al. Effects of disclosing financial interests on attitudes toward clinical research. J Gen Intern Med. 2008;23(6):860-6.

Weinfurt KP, Hall MA, Friedman JY, Hardy NC, Fortune-Greeley AK, Lawlor JS, et al. Effects of disclosing financial interests on participation in medical research: A randomized vignette trial. Am Heart J. 2008;156(4):689–97.

Wells KJ, McIntyre J, Gonzalez LE, Lee JH, Fisher KJ, Jacobsen PB, et al. Feasibility trial of a Spanish-language multimedia educational intervention. Clin Trials. 2013;10(5):767-74.

Welton AJ, Vickers MR, Cooper JA, Meade TW, Marteau TM. Is recruitment more difficult with a placebo arm in randomised controlled trials? A quasirandomised, interview based study. BMJ. 1999;318:1114-7.

Weston J, Hannah M, Downes J. Evaluating the benefits of a patient information video during the informed consent process. Patient Education and Counseling. 1997;30:239-45.

Witham MD, Band MM, Price RJG, Fulton RL, Clarke CL, Donnan PT, et al. Effect of two different participant information sheets on recruitment to a falls trial: An embedded randomised recruitment trial. Clin Trials. 2018;15(6):551-6.

Wong AD, Kirby J, Guyatt GH, Moayyedi P, Vora P, You JJ. Randomized controlled trial comparing telephone and mail follow-up for recruitment of participants into a clinical trial of colorectal cancer screening. Trials. 2013;14:40.
